# Prediction of gait recovery using machine learning algorithms in patients with spinal cord injury

**DOI:** 10.1097/MD.0000000000038286

**Published:** 2024-06-07

**Authors:** Hyun-Joon Yoo, Bummo Koo, Chan-woo Yong, Kwang-Sig Lee

**Affiliations:** a Korea University Research Institute for Medical Bigdata Science, Korea University College of Medicine, Seoul, Republic of Korea; b School of Health and Environmental Science, Korea University College of Health Science, Seoul, Republic of Korea; c AI Center, Korea University Anam Hospital, Korea University College of Medicine, Seoul, Republic of Korea.

**Keywords:** functional ambulation category, gait recovery, machine learning, prediction algorithm, spinal cord injury

## Abstract

With advances in artificial intelligence, machine learning (ML) has been widely applied to predict functional outcomes in clinical medicine. However, there has been no attempt to predict walking ability after spinal cord injury (SCI) based on ML. In this situation, the main purpose of this study was to predict gait recovery after SCI at discharge from an acute rehabilitation facility using various ML algorithms. In addition, we explored important variables that were related to the prognosis. Finally, we attempted to suggest an ML-based decision support system (DSS) for predicting gait recovery after SCI. Data were collected retrospectively from patients with SCI admitted to an acute rehabilitation facility between June 2008 to December 2021. Linear regression analysis and ML algorithms (random forest [RF], decision tree [DT], and support vector machine) were used to predict the functional ambulation category at the time of discharge (FAC_DC) in patients with traumatic or non-traumatic SCI (n = 353). The independent variables were age, sex, duration of acute care and rehabilitation, comorbidities, neurological information entered into the International Standards for Neurological Classification of SCI worksheet, and somatosensory-evoked potentials at the time of admission to the acute rehabilitation facility. In addition, the importance of variables and DT-based DSS for FAC_DC was analyzed. As a result, RF and DT accurately predicted the FAC_DC measured by the root mean squared error. The root mean squared error of RF and the DT were 1.09 and 1.24 for all participants, 1.20 and 1.06 for those with trauma, and 1.12 and 1.03 for those with non-trauma, respectively. In the analysis of important variables, the initial FAC was found to be the most influential factor in all groups. In addition, we could provide a simple DSS based on strong predictors such as the initial FAC, American Spinal Injury Association Impairment Scale grades, and neurological level of injury. In conclusion, we provide that ML can accurately predict gait recovery after SCI for the first time. By focusing on important variables and DSS, we can guide early prognosis and establish personalized rehabilitation strategies in acute rehabilitation hospitals.

## 1. Introduction and literature review

### 1.1. Introduction

Spinal cord injury (SCI) is a devastating disorder that causes abnormalities of bodily function below the level of injury due to sensory, motor, and autonomic dysfunctions. The worldwide incidence of traumatic SCI varies between countries, but the overall incidence is suspected to be 23.0 cases per million worldwide.^[1,2]^ Although the global epidemiology of nontraumatic SCI (e.g., degenerative myelopathy or neoplasm) is less robust, it has been reported to be increasing in developed countries.^[3]^ Since it often leads to extensive physical and emotional disabilities in patients, families, and even in society, accurate prediction of recovery is critical for healthcare professionals to enable decisions regarding the most suitable types of rehabilitation strategies and facilities.^[4]^

Many previous studies and systematic reviews have identified prognostic factors and algorithms for predicting functional outcomes.^[5,6]^ There has been extensive research regarding the prediction of gait recovery because gait dysfunction is a serious consequence of SCI and recovery of walking ability is of the highest priority for such patients. For example, van Middendorp et al^[7]^ developed a clinical prediction rule based on the age and clinical neurological parameters, such as motor and sensory scores, to predict the long-term probability of walking independently after traumatic SCI. Furthermore, combining neurophysiological data, such as motor and sensory evoked potentials, and nerve conduction studies with other clinical evaluations have been suggested to improve the prediction of ambulation capacity.^[8,9]^ Advances in neuroimaging studies have allowed for the investigation of the association between walking ability and various neuroimaging biomarkers, such as midsagittal tissue bridges and diffusion tensor imaging parameters.^[10,11]^

Machine learning (ML) algorithms are widely adopted in the medical field for various purposes, including disease diagnosis, drug discovery, and medical data analysis.^[12]^ In particular, ML has several advantages over traditional statistical methods (e.g., linear or logistic regression) in predicting and developing prognostic models of certain diseases. It has few restrictions on the number of predictors derived from a given dataset and is suitable for determining complex nonlinear relationships within datasets. Owing to these advantages, ML has also been increasingly applied in the predictive modeling of various outcomes following SCI (e.g., quality of life, duration of opioid prescription, duration of intensive care unit stay, and mortality).^[13–15]^ A few ML studies have been conducted regarding the prognostication of neurological outcomes. One retrospective study attempted to predict self-reported ambulation ability and functional independence using an artificial neural network.^[16]^ Another recent study developed an unsupervised ML algorithm that could predict walking function 1 year after the traumatic SCI. The study also demonstrated equivalent performance to that of the logistic regression model (see more details in Section 1.2.).^[17]^

Predicting gait ability at discharge from acute rehabilitation hospitals when establishing a discharge plan is important (e.g., transfer to a subacute inpatient rehabilitation facility or home discharge). It also is of paramount importance in designing rehabilitation strategies. For individuals who are expected to recover sufficiently with independent gait function, rehabilitation approaches mainly focus on restorative techniques, such as endurance training, balance training, and lower-extremity strengthening, to promote neuroplasticity and enhance independent gait. In contrast, for individuals with limited potential for neurorecovery, there is more emphasis on compensatory techniques, such as wheelchair mobility or bed transfer.^[18]^ However, despite its importance, studies on this topic have been lacking^[19]^ and most have focused on the final walking ability when neurologic recovery reaches a plateau.^[5–7]^ In particular, no study has employed a ML approach. In addition, previous studies have mainly focused on the effects of individual prognostic factors, and few studies have considered the interaction between each factor and optimized the modeling by considering the confounding factors between each variable.^[2]^

This retrospective study aimed to predict gait function at discharge from an acute inpatient rehabilitation facility following SCI using a ML algorithm. Since we aimed to analyze the integrated data of all kinds of SCI syndrome, admission data from both traumatic and non-traumatic SCI and cauda equina syndrome were integrated and analyzed. Considering that the clinical data area is complex and potentially nonlinear, we hypothesized that a ML approach would outperform conventional regression analysis. In addition, we explored important variables that were closely related to walking ability. Finally, we attempted to maximize the explainability of the ML approach by suggesting decision tree models that can be incorporated into clinical decision-making.

### 1.2. Literature review

ML has become a popular tool and contributed to improvement in diagnosis, risk assessment, and predicting prognosis.^[20]^ Accurate prediction of a disease outcome and establishing rehabilitation strategy to maximize the estimated functional gain is the one of the key component of rehabilitation medicine. As a result, studies regarding outcome prediction have been increasing in the field of SCI medicine. In this section, we summarized recent previous ML studies in the SCI medicine for outcome prediction (Table 1). Especially, we focused on the studies regarding prediction of functional gain which is the main topic of this article. Therefore, other research topics such as prediction of mortality, opioid usage, and pressure ulcers are excluded in the review.

**Table 1:** Summary of recent literature review of ML study in neurological outcome prediction after spinal cord injury.

Author	Year	Type of ML	Purpose and summary of the study
Belliveau et al^[16]^	2016	Artificial neural network	Predict functional outcome (self-reported ambulation ability and FIM) 1 yr after traumatic SCI
DeVries et al^[17]^	2020	Unsupervised ML, linear regression	Prediction of walking recovery (FIM) following SCI at discharge or 1 ≥yr follow-up
Facchinello et al^[21]^	2021	Regression tree	Functional outcome prediction (SCIM) within the first-year post-injury
Torres-Espin et al^[22]^	2021	Topological extraction, logistic regression	Predict neurological recovery measured by AIS grade at discharge from the hospital on the basis of mean arterial pressure during surgery
Okimatsu et al ^[23]^	2022	Deep learning-based radiomics and convolutional neural network	Determine the functional prognosis of patients (AIS grade) assessed at 1 mo after injury with cervical SCI based on MRI findings
Chihiro et al ^[24]^	2024	Ensemble ML	Predict functional outcomes (SCIM) with features present at the time of rehabilitation admission

AIS = American Spinal Injury Association Impairment Scale, FIM = functional independence measure, ML = machine learning, MRI = magnetic resonance image, SCI = spinal cord injury, SCIM = spinal cord independence measure.

As a result, a total of 6 studies were reviewed.^[16,17,21–24]^ All were retrospective designed and the etiology of the subjects was trauma. Two studies focused on walking ability prediction measured by Functional Independence Measure, and other 2 studies predicted functional independence using Spinal Cord Independence Measure, and the others tried to predict AIS grade after injury. Three studies tried to predict acute prognosis of neurological outcome (AIS grade,^[23]^ Spinal Cord Independence Measure^[22,24]^ at 1 month after injury or at the time of discharge. However, no study has focused on gait recovery at the early rehabilitation phase. Moreover, all the studies were conducted with patients with traumatic SCI.

## 2. Materials and methods

### 2.1. Participants

This retrospective study included data of patients with acute SCI who were admitted to the acute rehabilitation facility of the Department of Physical Medicine and Rehabilitation in Korea University Anam Hospital, Seoul, Republic of Korea, during June 2008 to December 2021. Patients’ demographic data and study variables were retrospectively reviewed by clinical research coordinators who were blinded to the study. From the dataset, we extracted data for all adult (≥18 years) patients with acute SCI, including cauda equina syndrome. Then, we excluded who were admitted for more than 120 days for acute care before rehabilitation. We assumed that such a long period of acute care might be a risk factor (e.g., very severe SCI combined with other organ or musculoskeletal injury) that prohibit active rehabilitation and considered it as an outlier.

Finally, patients with complete medical information on the study variables were included. This study was approved by the Institutional Review Board of Korea University Anam Hospital (2022AN0473). The requirement for informed consent was waived by the institutional review board.

### 2.2. Variables

The dependent variable was walking ability at discharge in an acute rehabilitation setting, as measured using the functional ambulation category (FAC), a clinical-based assessment that distinguishes 6 levels of walking ability based on the amount of physical support required (0–5; 0 indicates inability to walk and 5 indicates walking independently).^[25]^

Independent variables were selected on the basis of previous literature indicating the possibility of the correlation with functional recovery after SCI and the availability of the retrospective data set.^[2,7,26,27]^ Especially, we tried to add as many variables as possible to maximize the strength of ML algorithms. Among them, we selected variables that can be easily assessed or routinely evaluated in most of the SCI clinics so that the study results can be widely applied to other institutions. We excluded laboratory and magnetic resonance image findings since it can be affected by various conditions and difficult to interpret any changes compared to pre-injury state. The selected independent variables were as follows: age; elderly (yes, defined as ≥65 years or no); sex; periods of acute care, defined as admission dates for acute medical or surgical management before transfer to rehabilitation ward; periods of rehabilitation, defined as admission dates in the rehabilitation ward; etiology of SCI (traumatic or nontraumatic), concomitant cardiovascular disease (CVD) (yes or no); diabetes mellitus (DM) (yes or no); and all neurological information entered on the International Standards for Neurological Classification of SCI worksheet: neurological level of injury (NLI; cervical, thoracic, lumbar or cauda equina), American Spinal Injury Association/International Spinal Cord Society neurological standard scale (AIS) grades, injury completeness (yes or no), motor scores, light touch and pinprick scores, voluntary anal contraction, and anal sensation.^[28]^ Presence of neurogenic bladder after SCI, and initial FAC were also included. Furthermore, we added the somatosensory-evoked potentials (SSEP) of the lower extremity data as input variables. The SSEP data were categorized into 3 groups: normal, prolonged, or shallow waveforms, and no response. All independent variables were recorded within the first 3 d after admission to the rehabilitation ward. We applied ML algorithms using these independent variables as inputs to predict FAC at discharge (FAC_DC).

### 2.3. ML analysis

Conventional linear regression analysis and 3 ML approaches were compared for the prediction of FAC_DC: random forest (RF), decision tree (DT), and support vector machine. A DT consists of internal nodes (the tests of independent variables), branches (the outcomes of the tests) and terminal nodes (the values of the dependent variable). A RF consists of many DTs, which take a majority vote on the dependent variable (“bootstrap aggregation”). A support vector machine creates a line or space (“hyperplane”), which separates data with the maximal distance between different groups. These 3 models were adopted because they are among the most popular machine-learning models.^[29,30]^

The study samples were split into training and test sets with a 75:25 ratio. The performance of each algorithm was evaluated using the root mean squared error (RMSE), the square root for the average of the squares of errors among the test set. The unit of the RMSE is the unit of the dependent variable, FAC_DC. Since the total number of data is not enough and data splitting in limited data for validation set might make a trained model unstable, we created a new data set from the existing data using random splitting and average technique. More specifically, the random split and analysis were repeated 10 times, and the average was used for external validation.

The maximum depth was not predetermined for the decision tree. The number of trees was 1000 for the random forest. Weight optimization was based on the limited-memory Broyden–Fletcher–Goldfarb–Shanno algorithm and each of 2 hidden layers had 10 neurons in the artificial neural network.

RF variable importance (residual-sum-of-squares decrease, averaged over 500 decision trees, from the creation of a branch on a certain predictor) was introduced for identifying the most important predictors of FAC_DC. For example, let us assume that the random forest variable importance of initial FAC for the prediction of FAC_DC for 353 participants is 180.45. This indicates that the residual sum of squares for 353 participants (averaged over 500 decision trees) decreases by 180.45 in case a branch is created by initial FAC. In other words, the RMSE (averaged over 500 decision trees) decreases by 0.71 in case a branch is created by initial FAC.

### 2.4. Decision support system

Finally, the DT in the 10th run (last run) was derived as a decision support system (DSS) for FAC_DC for the clinical implication. The DSS was developed for each of 3 groups, that is, all patients, those with trauma and those with non-trauma. As to be seen later, the DT based DSS is expected to show 3 notable strengths compared to traditional statistical approaches such as linear regression. Firstly, it can deal with and classify both categorical and numerical variables and simple to comprehend. Secondly, it logically presents variables that should be prioritized in the clinical decision-making process. Especially, it is capable of handling variable interactions in terms of sequential information.^[31]^ Thirdly, it can guide the effective cutoff points of a continuous predictor for different groups (e.g., initial FAC < 2 in the trauma group vs. initial FAC < 1 in the non-trauma group regarding good clinical outcome). All analyses were conducted in November 2021 using R-Studio 1.3.959 (R-Studio Inc., Boston, MA).

## 3. Results

### 3.1. Demographics of the participants

**Figure 1.**
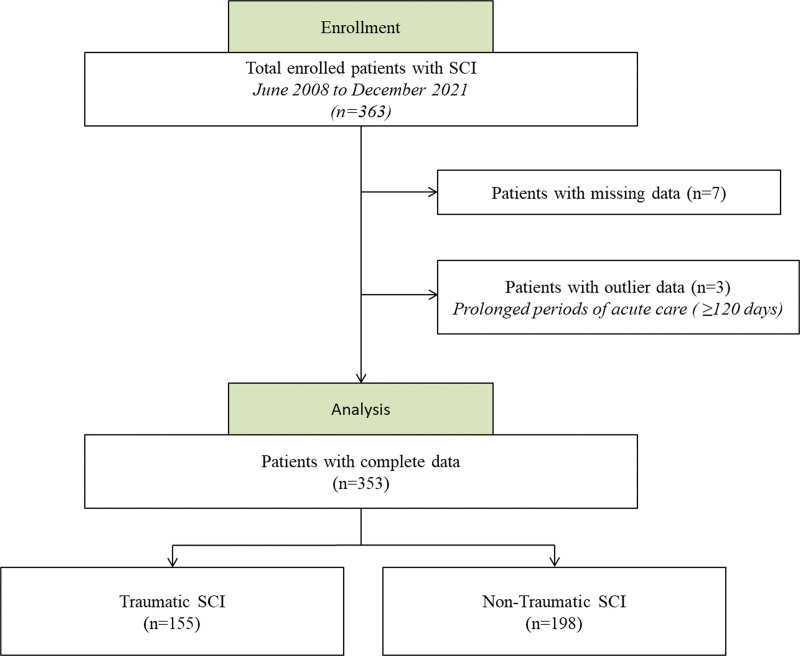
Flowchart of the data enrollment. A total of 353 patients were finally included on the following process.

A total of 363 adult patients with acute SCI were enrolled in this study, and their medical records were retrospectively reviewed. Among them, patients with missing clinical data (n = 7) and outlier data (n = 3) were excluded in the final analysis. As a result, 353 patients (155 with traumatic SCI and 198 with nontraumatic SCI) with complete information were included in the study (Fig. 1). Descriptive statistics for all participants, including those with and without trauma, are presented in Table 2. Participants with nontraumatic SCI tended to be older than those with traumatic injury and were more likely to have concomitant CVD or DM. In the case of traumatic SCI, the male sex ratio was relatively higher (76.5%) compared to that of non-traumatic SCI (59.1%). In addition, the periods of acute care were longer in the nontraumatic group, probably because of other comorbidities that could affect admission dates. The other initial demographic and neurological data were similar between the traumatic and nontraumatic SCI groups. Overall, AIS grade D was the most common impairment scale in all groups and about half of patients injured cervical cord. Prolonged or shallow waveforms were the most commonly observed SSEP findings. The mean initial FAC score was around 1, suggesting most of the patient required maximal support for ambulation. On the other hand, the mean FAC_DC score was around 2, indicating most of patients need less support after adequate rehabilitation.

**Table 2:** Demographic and neurological information of the study participants.

Variables	All participants (n = 353)	Participants with traumatic SCI (n = 155)	Participants with non-traumatic SCI (n = 198)
Age at injury (yr)	61.39 ± 16.48	56.75 ± 16.74	65.02 ± 15.37
Elderly (≥65 yr); n (%)	172 (48.7)	54 (34.8)	118 (59.6)
Sex (male/female)	231/122	114/41	117/81
Periods of acute care (d)	63.19 ± 62.83	43.57 ± 53.39	78.55 ± 65.46
Periods of rehabilitation (d)	30.04 ± 13.25	30.6 ± 15.63	29.6 ± 19.58
CVD, n (%)	175 (49.6)	64 (41.3)	111 (56.1)
DM, n (%)	86 (24.4)	29 (18.7)	57 (28.8)
AIS grades, n (%)			
Grade A	29 (8.2)	22 (14.2)	7 (3.5)
Grade B	18 (5.1)	11 (7.1)	7 (3.5)
Grade C	53 (15.0)	30 (19.4)	23 (11.6)
Grade D	251 (71.1)	92 (59.3)	159 (80.4)
Grade E	2 (0.6)	0 (0.0)	2 (1.0)
NLI, n (%)			
Cervical	195 (55.2)	113 (72.9)	82 (41.4)
Thoracic	101 (28.6)	27 (17.4)	74 (37.4)
Lumbar or cauda equina	57 (16.2)	15 (9.7)	42 (21.2)
SSEP, n (%)			
Normal	77 (21.8)	34 (21.9)	43 (21.7)
Prolonged or shallow	149 (42.2)	61 (39.4)	88 (44.4)
No response, n (%)	127 (36.0)	60 (38.7)	67 (33.9)
Initial FAC	1.224 ± 1.42	1.123 ± 1.53	1.303 ± 1.33
FAC_DC	2.176 ± 1.61	2.052 ± 1.76	2.273 ± 1.47

Data are expressed as mean ± standard deviation or n unless otherwise indicated.

AIS = American Spinal Injury Association Impairment Scale, DM = diabetes mellitus, FAC = functional ambulation categories, FAC_DC = functional ambulation categories at the time of discharges, NLI = neurological level of injury, SCI = spinal cord injury, SSEP = somatosensory evoked potential.

### 3.2. Model performance

The performance measures (RMSEs) of each model for FAC_DC are listed in Table 3. Three sets of models were considered for 3 groups of participants: all participants, those with trauma, and those without. The performance measures of the RF and the DT showed the best performance: 1.09 and 1.24 for all participants, 1.20 and 1.06 for those with trauma, and 1.12 and 1.03 for those with nontrauma, respectively. The conventional linear regression analysis showed high RMSE, especially with a small data subset (1.30 for all participants, 2.43 for those with trauma, and 2.12 for those with nontrauma)

**Table 3:** Model performance using root mean squared error.

	All participants (n = 353)	Participants with traumatic SCI (n = 155)	Participants with non-traumatic SCI (n = 198)
Random forest	1.09	1.20	1.12
Decision tree	1.24	1.06	1.03
Support vector machine	1.28	1.32	1.09
Linear regression	1.30	2.43	2.12

SCI = spinal cord injury

### 3.3. Important variables

The RF variable importance results for the 10th run (the last run) are presented in Table 4. The 10 most significant predictors of FAC_DC for all participants were the initial FAC, lower-extremity motor scores (bilateral hip flexor, bilateral ankle dorsiflexor, and left knee extensor), NLI, age, periods of acute care, and periods of rehabilitation. Although the rankings of the important variables were somewhat different, the selected important variables were similar between the analyses of patients with traumatic and nontraumatic SCI. Especially initial FAC score was found to be the most important variable across all groups. For instance, the residual sum of squares of initial FAC (180.45) was much higher than that of the 2nd important variable, NLI (52.97) in all participants group. Sensory scores entered on the International Standards for Neurological Classification of SCI worksheet, concomitant medical diseases (CVD or DM) and SSEP were not ranked among the 10 most important variables.

**Table 4:** Variable importance for the prediction of FAC_DC.

	All participants (n = 353)	Participants with traumatic SCI (n = 155)	Participants with non-traumatic SCI (n = 198)
1st	Initial FAC (180.45)	Initial FAC (78.69)	Initial FAC (62.57)
2nd	NLI (52.97)	Hip Flexor, Lt. (41.04)	NLI (38.42)
3rd	Ankle Dorsiflexor, Lt. (49.13)	Long Toe Extensor, Lt. (21.84)	AIS Grade (19.16)
4th	Hip Flexor, Lt. (39.87)	Ankle Dorsiflexor, Lt. (17.79)	Knee Extensor, Rt. (18.27)
5th	Hip Flexor, Rt. (26.85)	NLI (16.33)	Age (16.42)
6th	Knee Extensor. Lt. (24.07)	Ankle Dorsiflexor, Rt. (12.07)	Periods of rehabilitation (13.22)
7th	Age (23.48)	Hip Flexor, Rt (11.17)	Ankle Plantar Flexor (11.49)
8th	Periods of acute care (22.84)	Knee Extensor. Lt. (10.99)	Hip Flexor, Rt (11.21)
9th	Periods of rehabilitation (21.97)	Age (10.47)	Ankle Dorsiflexor, Rt. (8.63)
10th	Ankle Dorsiflexor, Rt. (21.75)	Periods of acute care (9.89)	Periods of acute care (7.66)

FAC = functional ambulation categories, FAC_DC = functional ambulation categories at the time of discharges, NLI = neurological level of injury, SCI = spinal cord injury.

*The residual sum of squares for 353 participants (averaged over 500 decision trees) decreases by 180.45 in case a branch is created by initial FAC. In other words, the root mean squares of errors (averaged over 500 decision trees) decrease by 0.71 in case a branch is created by the initial FAC.

### 3.4. Decision support system based on the decision trees

**Figure 2.**
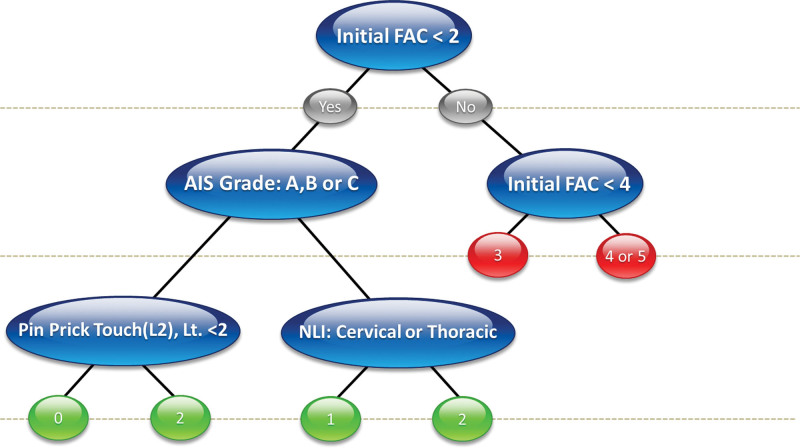
Decision support system of all patients with spinal cord injury. Initial FAC is the key variable on the decision support system. FAC = functional ambulation categories.

**Figure 3.**
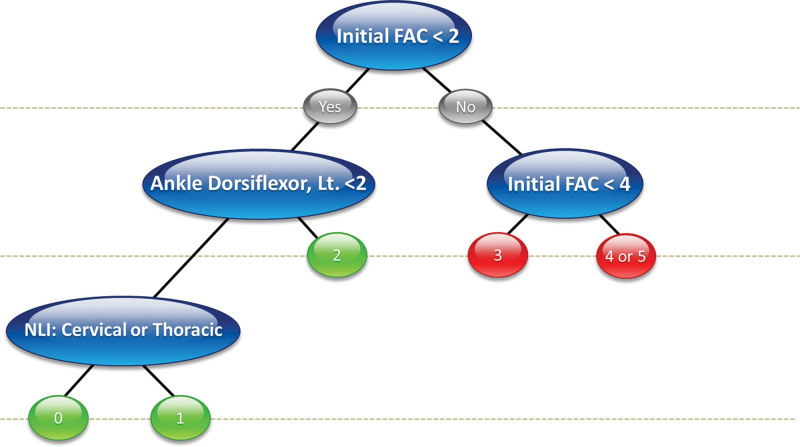
Decision support system of patients with traumatic spinal cord injury. Initial FAC is the key variable on the decision support system. FAC = functional ambulation categories.

**Figure 4.**
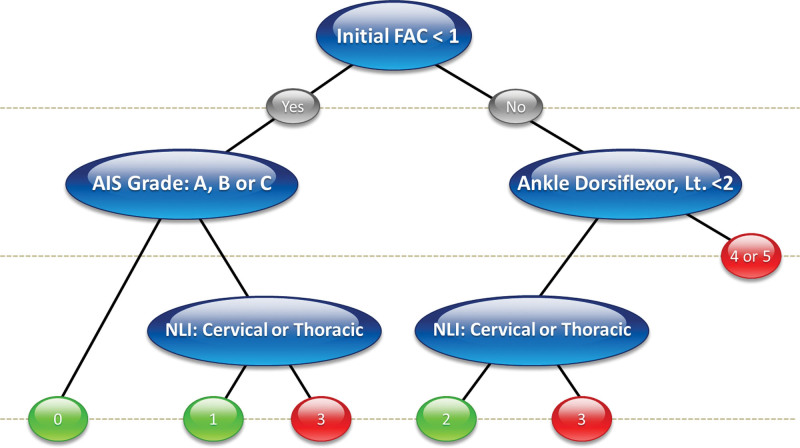
Decision support system of patients with non-traumatic spinal cord injury. Please note that if the AIS grade was D or E and the NLI was at the lumbar level, the patients had the possibility of recovering gait function to FAC 3, even if they were FAC 0 at the time of admission. AIS = American Spinal Injury Association Impairment Scale, FAC = functional ambulation categories, NLI = neurological level of injury.

The DSS based on the DT in the 10th run (last run) is presented in Figures 1–3. The figure represents the DSS of all patients (Fig. 2), those with trauma (Fig. 3), and those without trauma (Fig. 4). The initial FAC was the most influential factor in all groups which is concordant with variable importance analysis. In addition, AIS grades A–C and NLI at the cervical or thoracic level negatively affected gait recovery in all groups. As shown in Figure 2, we expected that patients with an initial FAC level below 2 would still have gait disturbance at the time of discharge even if they are AIS grade D or E; FAC_DC would be 0, 1, or 2. This indicates that the initial gait function is the most important factor in gait recovery. In particular, an initial FAC < 2 and AIS grades A, B, or C with impaired pinprick touch on the L2 segment are likely to have a poor prognosis.

In Figure 3, which describes DSS for traumatic SCI, the patients with an initial FAC level below 2 with ankle dorsiflexor weakness and NLI in the cervical or thoracic region showed the worst outcome (FAC 0). Like the DSS of all patients, initial FAC score 2 was the most important cutoff point for predicting FAC_DC, followed by ankle dorsiflexion weakness and NLI (cervical or thoracic versus lumbar). If the initial FAC score is less than 2, they will still need support at the discharge (FAC_DC will be 0,1 or 2) and not be able to gait independently.

In patients with nontraumatic SCI, FAC 1 was the most important cutoff point. Patients with an initial FAC value of < 1 with AIS grades A to C and NLI in the cervical or thoracic region were less likely to recover gait function at the time of discharge. Interestingly, if the AIS grade was D or E and the NLI was at the lumbar level, the patients had the possibility of recovering gait function to FAC 3, even if they were FAC 0 at the time of admission. These findings indicate that not only initial FAC but also the AIS grade is important in predicting the gait recovery in nontraumatic SCI group. Especially, active rehabilitation for independent gait is important in lumbar SCI patients with AIS grade D or E.

## 4. Discussion

Predicting gait recovery after SCI is paramount for an effective rehabilitation strategy; however, this remains challenging. In this study, we demonstrated that ML models can accurately predict gait function at discharge from acute rehabilitation hospitals in patients with acute SCI. Through the ML algorithm, we could predict FAC_DC more accurately and with good performance by combining demographic features and neurologic evaluation at admission to an acute rehabilitation facility. In addition, we identified variables on admission, such as initial FAC, lower extremity motor scores, and NLI, as strong predictors of gait function recovery. In particular, using a simple DSS, we developed a sequential decision algorithm that can be applied in a clinical setting. To the best of our knowledge, this is the first ML study to predict gait function at discharge from an acute rehabilitation hospital in patients with SCI.

The FAC is a widely used clinical gait assessment scale that categorizes walking disability according to dependency.^[25]^ Since it is a reliable, valid, and convenient tool to evaluate walking ability at clinical admission, the authors’ institution assesses the gait function of hospitalized patients using FAC routinely. Previous studies also have used FAC to measure gait function in patients with SCI.^[32,33]^ It is not only known to be correlated with walking velocity and step length,^[34]^ but is also often used for treatment and transfer planning during the stay in the rehabilitation setting.^[35]^ Therefore, early prediction of FAC at discharge is fundamental in establishing rehabilitation strategies through accurate goal setting. Depending on the expected gait function, home discharge or transfer to a subacute rehabilitation facility where intensive inpatient care is possible can be planned. In addition, by guiding achievable goals, maximizing patients’ motivation and engagement in rehabilitation programs is possible.^[36]^

In this study, RF and DT performed better and predicted FAC_DC more accurately than conventional linear regression. RF and DT have gained massive popularity in various ML fields because of their good ability in classification and prediction.^[37,38]^ In particular, they have been applied in statistically challenging settings in which the number of variables is high.^[39]^ To make use of the advantages of ML, we included as many demographic and neurophysiological variables as possible. We also included variables related to time, such as admission dates for acute care and admission dates in the rehabilitation ward. These time-related variables are expected to have strong interactions with other independent variables, and RT and DT seem to effectively analyze nonlinear and complex medical records.

Previous studies have evaluated the predictive power of neurological variables on ambulation outcomes. van Middendorp et al^[7]^ suggested that age and motor- and light touch scores of L3 and S1 were the best predictors of 1-year ambulation outcomes after traumatic SCI. Another study on traumatic SCI indicated that the AIS grades and age were highly correlated with walking function at discharge.^[19]^ Although some variables were not included in this study, a recent ML study mentioned age, AIS grades, concomitant degenerative spine diseases and some radiographic information; these were highly correlated with functional motor status 6 months after traumatic cervical SCI.^[40]^ In our study, the initial FAC value was the most important variable predicting FAC_DC in both traumatic and nontraumatic SCI, indicating that the initial gait function is the most important factor in the prognosis. NLI, AIS grades, and age were also designated as important predictors, in keeping with previous studies.^[7,19,40–42]^ In a previous study, SSEP was known to be related to the outcome of ambulatory capacity.^[43]^ Even though the SSEP was not selected as one of the 10 most significant predictors in this study, it was ranked as the 15th, 12th, and 11th most important predictor in the analysis of all patients, patients with traumatic SCI, and those with nontraumatic SCI, respectively. Therefore, we believe that evaluation of SSEP provides additional diagnostic value for the assessment of acute SCI. Although the order of variable importance differed slightly, the overall results were similar between the traumatic and nontraumatic SCI groups.

The main strength of this study is that we proposed a DT-based DSS that aids in the prediction of FAC_DC. The most notable characteristic of DT is its ability to predict the dependent variable by learning simple decision rules inferred from the feature set.^[38]^ Unlike previous studies, which summated values of relevant variables to predict target outcomes,^[7,14,44]^ we provided sequential decision rules based on key predictors. Such a sequential algorithm would guide clinical decision making more logically. In addition, we attempted to predict multiclass outcomes, FAC 0 to 5, which is statistically more challenging than previous binary classification tasks.^[7,14,44]^ In the DSS results, the common key predictors were initial FAC and NLI. In particular, it was found that the initial FAC, which was also the most important variable in the Gini analysis, had a significant influence on discharge planning. Referring the DSS derived from all patients group (Fig. 2), patients with an FAC value <2 still needed assistance in walking, even at the time of discharge (FAC 0–2), indicating that they still required further inpatient rehabilitation. In particular, if a patient’s initial FAC score was less than 2 and AIS grade is A, B or C with impairment of pin prick touch at L2 dermatome, the patient will hardly gain gait recovery. In that case, early adaptation of compensatory techniques can be benefit. In contrast, patients with a FAC value ≥2 could consider home discharge or at least continue rehabilitation at outpatient clinic because they were likely to need little assistance (FAC 3) or recover to independent gait (FAC 4 or 5). Interestingly, there was a subgroup that can show excellent prognosis even if the initial FAC score is 0 in non-traumatic SCI group (Fig. 4). If a patient NLI is D or E and the NLI is lumbar lesion, the patient will recover to FAC score 3. This is quite contradictory result to that of traumatic SCI group, which has poor prognosis when the initial FAC score is less than 2. Although the suggested DSS might require further revision, an initial evaluation based on the suggested predictors can guide the rehabilitation strategy and discharge planning. However, care must be taken since uncertainty and imprecision are inherent in modeling clinical real-world.

A number of studies have investigated walking outcomes after traumatic SCI, while studies with nontraumatic SCI are still scarce.^[45]^ In contrast, we analyzed 2 groups with different etiologies and proposed DSS that integrated both traumatic and nontraumatic SCI in order to maximize clinical utility. Another strength of this study is that the retrospective data were reliable because the dependent and independent variables were assessed by well-trained physicians and recorded appropriately. Despite these strengths, this study had some limitations. First, because the study data were generated from a single unbalanced cohort, the predictive ability may not be generalizable to other institutions. Therefore, it is uncertain whether the suggested ML algorithms and DSS will show similar results in other cohort. From this perspective, the decision boundaries (e.g., FAC score 2 in DSS of all patients group) or variable importance can be changed in other data set. Additionally, the cohort might be imbalanced (e.g., the NLI and AIS grades are not evenly distributed) and it would negatively affect the reproducibility of the study results. Second, the sample size may have been relatively small for ML research. We concluded the sample size was not enough to construct a validation set. Instead, we used internal validation with data splitting and averaging. For these reasons, multi-cohort modeling with larger data set and external validation are needed to advance the model. Finally, applying the study results, including the DSS model, in actual practice is essential to determine the clinical usefulness and feasibility of the study.

## 5. Conclusion

Early prediction of gait ability after SCI and the establishment of a rehabilitation strategy are the most important steps before beginning acute rehabilitation. Besides, ML has considerable predictive accuracy and is a promising prediction tool for various conditions. Here, we reported the possibility that ML, especially RT- and DT-based models, could accurately predict the degree of gait recovery using clinical data at the time of admission to an acute rehabilitation facility. Additionally, this study demonstrates the strength of ML as an explainable artificial intelligence for identifying the most important predictors. Finally, we provided a DSS based on the DT for a more intuitive understanding and suggest clinical applicability of ML in practice. This is the first to demonstrate simple DSS based on ML approach which can be directly applied to real- world. This cutting-edge approach provided insights that early prediction can be achieved with clinical features at the timing of admission. In conclusion, the results of this study could guide optimal rehabilitation plans based on realistic therapeutic goals and optimize the efficiency and efficacy of treatment strategies. This study highlights the importance of ML application and could be the cornerstone of future SCI rehabilitation research in predicting prognosis, diagnosis, and personalized treatment. We believe that this study will contribute to the best-practice guidelines for managing patients with acute SCI.

## Author contributions

**Conceptualization:** Hyun-Joon Yoo, Kwang-Sig Lee.

**Data curation:** Hyun-Joon Yoo, Bummo Koo, Chan-woo Yong, Kwang-Sig Lee.

**Formal analysis:** Bummo Koo, Chan-woo Yong, Kwang-Sig Lee.

**Funding acquisition:** Hyun-Joon Yoo.

**Investigation:** Hyun-Joon Yoo, Kwang-Sig Lee.

**Methodology:** Hyun-Joon Yoo, Bummo Koo, Kwang-Sig Lee.

**Project administration:** Hyun-Joon Yoo.

**Resources:** Hyun-Joon Yoo.

**Software:** Kwang-Sig Lee.

**Supervision:** Hyun-Joon Yoo, Kwang-Sig Lee.

**Validation:** Kwang-Sig Lee.

**Visualization:** Bummo Koo, Chan-woo Yong, Kwang-Sig Lee.

**Writing – original draft:** Hyun-Joon Yoo, Kwang-Sig Lee.

**Writing – review & editing:** Hyun-Joon Yoo, Kwang-Sig Lee.
